# Effective treatment of heavy and/or prolonged menstrual bleeding without organic cause: pooled analysis of two multinational, randomised, double-blind, placebo-controlled trials of oestradiol valerate and dienogest

**DOI:** 10.3109/13625187.2011.591456

**Published:** 2011-07-20

**Authors:** Ian S Fraser, Susanne Parked, Uwe Mellinger, Andrea Machlitt, Marco Serrani, Jeffrey Jensen

**Affiliations:** * University of Sydney, Sydney, New South Wales, Australia; † Global Clinical Development, Bayer Pharma AG, Berlin, Germany; ‡ Global Medical Affairs Women's Healthcare, Bayer Pharma AG, Berlin, Germany; § Oregon Health & Science University, Portland, OR, USA

**Keywords:** Oral contraceptive, Heavy menstrual bleeding

## Abstract

**Objectives** To evaluate the efficacy of oestradiol valerate/dienogest (E_2_V/DNG) for the treatment of heavy and/or prolonged menstrual bleeding without organic pathology based on the analysis of data from two identically designed double-blind, randomised studies. **Methods** Women aged ≥ 18 years with heavy and/or prolonged menstrual bleeding were randomised to E_2_V/DNG (*n* = 269) or placebo (*n* = 152) for 196 days. Objective changes in menstrual blood loss (MBL) volume were assessed using the alkaline haematin method. **Results** After six months of treatment, median MBL decreased by 88% with E_2_V/DNG compared with 24% with placebo. The greatest reduction was achieved at the first withdrawal bleed after treatment initiation and it was sustained with no loss of effect throughout treatment. **Conclusion** E_2_V/DNG was more effective than placebo in reducing MBL in women with heavy and/or prolonged menstrual bleeding without organic pathology. The reduction was largely achieved as early as the first withdrawal bleed, with further gradual improvement throughout treatment.

## INTRODUCTION

Abnormal uterine bleeding encompasses abnormalities in the regularity, duration of flow, frequency, and/or blood fl ow volume relative to ‘normal’ menstruation^1^. Of these menstrual abnormalities, heavy menstrual bleeding, defi ned objectively as blood loss ≥ 80 ml per menstrual cycle^2^, is a common clinical problem accounting for approximately 12% of all gynaecological referrals^3^. Heavy menstrual bleeding can have an adverse impact on women's health-related quality of life and is an important cause of iron deficiency anaemia^4^. Population-based estimates based on objective determination of monthly menstrual blood loss (MBL) suggest that blood loss > 80 ml per menstrual cycle occurs in about 9–14% of menstruating women, while the prevalence based on subjective perceptions ranges between 20 and 52%^4^.

Both surgical and medical interventions are available for the treatment of heavy menstrual bleeding. Treatments include the levonorgestrel-releasing intrauterine system (LNG-IUS), tranexamic acid, non-steroidal anti-inflammatory drugs, danazol, cyclical progestogens, and combined oral contraceptives (COCs)^5^.With the exception of the LNG-IUS, cyclical progestogens and tranexamic acid, most medical treatments are prescribed off-label and without prospective, well-designed studies that validate or quantify their effect. Although COCs are used widely ‘off-label’ for the treatment of heavy or prolonged menstrual bleeding, prior to 2009, no large-scale objective, randomised or placebo-controlled data existed in the literature to support their use for this purpose^6^, with only one small randomised trial reported^7^. Surgical procedures such as hysterectomy and endometrial ablation offer effective strategies, but their cost and associated morbidity should limit these approaches to patients with whom medical therapies have failed^8^.

A novel and effective oral contraceptive containing oestradiol valerate and dienogest (E_2_V/DNG) has been shown to be associated with good cycle control and a good tolerability profile^9^,^10^. This oral contraceptive has been available since May 2009 in many countries worldwide, under the trade names Qlaira® or Natazia™. Recently, two identically designed Phase III trials, one conducted in Europe and Australia and the other in North America, were undertaken to evaluate the efficacy and safety of E_2_V/DNG in the treatment of heavy and/or prolonged menstrual bleeding^11^,^12^. This paper details the reduction in MBL and safety findings as well as the patients' and investigators' rating of the improvement in bleeding symptoms from an analysis of pooled data from these two Phase III clinical trials. By pooling data from the available studies, we sought to better define the decrease in MBL achieved with E_2_V/DNG and its safety profile across a larger and more diverse population of women. A pooled analysis of these trials was judged to be appropriate because of their identical design and enrolment criteria.

## METHODS

### Study design and participants

This is a pooled analysis of two randomised, double-blind, placebo-controlled trials conducted to investigate the efficacy and safety of E_2_V/DNG for the treatment of heavy and/or prolonged menstrual bleeding. One study was conducted in the USA and Canada between December 2005 and May 2008 (ClimcalTnals.gov Identifier: NCT00293059), while the other was conducted in Australia and Europe (Czech Republic, Finland, Germany, Hungary, the Netherlands, Poland, Sweden, the United Kingdom and Ukraine [ClinicalTrials.gov Identifier: NCT00307801]) between February 2006 and May 2008. Both studies had identical designs and analysis plans; full details have been published previously^11^,^12^.

In brief, the studies consisted of four parts: a screening phase of 28 days, a 90-day run-in phase (to confirm symptoms of heavy, prolonged and/or frequent bleeding), a 196-day treatment phase (which concluded with a 90-day efficacy phase that had to start on the first day of a treatment cycle), and a follow-up phase of 30 days. A 90-day reference period was chosen in accordance with the World Health Organisation (WHO) recommendations. This duration has been widely used for several decades to assess bleeding patterns with hormonal therapies^13^.

To be eligible to participate in the study, women had to be aged 18 years or older and have symptoms of heavy, prolonged and/or frequent menstrual bleeding (confirmed during the 90-day run-in phase) without ‘organic cause’ (i.e., no lesion in the reproductive tract such as uterine fibroids, adenomyosis, endometrial polyps, severe hyperplasia or malignancy). Women had to be willing to use a barrier method of contraception (since the preparation had not been approved as a contraceptive prior to the start of these studies and because these were double-blind studies) and to use and collect sanitary protection items (pads and tampons) provided by the sponsor for the duration of the study. Full details of the inclusion and exclusion criteria have been published previously^11^,^12^. For ethical reasons, use of iron supplements in these studies was allowed if considered necessary by the attending physician.

### Study treatment

Following the 90-day run-in phase, women with confirmed excessively heavy (two or more bleeding episodes each with a blood loss volume of 80 ml or more), prolonged (two or more bleeding episodes each lasting eight or more days) and/or frequent (more than five bleeding episodes with 20 or more bleeding days overall) menstrual bleeding were randomised to the E_2_-V/DNG oral contraceptive (Qlaira®/Natazia™; Bayer Pharma AG, Berlin, Germany) or matching placebo for 196 days (seven treatment cycles, each of 28 days). Study medication was started on the first day of bleeding following randomisation, and taken daily with no tablet-free days between cycles. The E_2_V/DNG oral contraceptive regimen was designed to deliver the oestrogen in a step-down and the progestogen in a step-up manner through each 28-day treatment cycle (E_2_V 3 mg on days 1-2, E_2_V 2 mg/DNG 2 mg on days 3–7, E_2_V 2 mg/DNG 3 mg on days 8–24, E_2_V 1 mg on days 25–26, and placebo on days 27–28). The treatment phase concluded with a 90-day efficacy phase.

### Study assessments

Throughout the study (including during the 90-day run-in phase), women were required to complete electronic diaries on a daily basis to document menstrual bleeding, the number of items of sanitary protection used, and study drug intake (during the treatment phase). Women were required to collect their used sanitary protection items (pads and tampons) in provided containers for objective analysis of MBL using a modified version of the alkaline haematin method^14^. A central laboratory was used to process and analyse all sanitary protection items.

### Study endpoints

#### Efficacy

The efficacy variables assessed in this pooled analysis were changes in MBL volume, the number of sanitary protection items used, and iron metabolism parameters.

#### Safety and tolerability

All women were given the opportunity to report adverse events associated with treatment. All spontaneously volunteered adverse events were documented. Additional safety assessments included the evaluation of vital signs, physical and gynaecological examinations, and mammography

### Statistical analysis

All variables were analysed based on the intent-to-treat (ITT) population (i.e., all randomised patients). MBL variables were also analysed in the subgroup of women with excessively heavy menstrual bleeding.

MBL was analysed during both the 90-day run-in phase and 90-day efficacy phase (which started on day 1 of treatment cycle 4 and ended on day 6 of treatment cycle 7). Bleeding episodes that started during a 90-day run-in/efficacy phase or cycle were counted completely within that period independently of whether the end of that bleeding episode was after the end of that phase/ cycle. Missing MBL data on non-consecutive days were replaced using the highest intensity value for bleeding obtained on the bordering days. No more than nine non-consecutive days were replaced per 90-day interval. Consecutive days with missing bleeding intensity data were not replaced.

The proportion of patients with improved menstrual bleeding symptoms as rated by investigators using the global assessment scale and by the patients using the patient's overall assessment scale was determined at the end of cycle 7 (when both investigators and patients were still blinded to treatment).

Safety outcomes were assessed in all randomised patients who took at least one dose of study medication. All variables were summarised using descriptive statistical methods. AN(C)OVA models were used to compare the treatment groups with the terms of treatment, pooled centre and baseline as covariates. A significance level of 0.05 was used. The mean, standard deviation, minimum, median and maximum were calculated for metric data. The change in MBL from the 90-day run-in phase to the 90-day efficacy phase was calculated along with the corresponding two-sided 95% confidence interval (CI). Frequency tables were generated for categorical data.

## RESULTS

**Figure 1:**
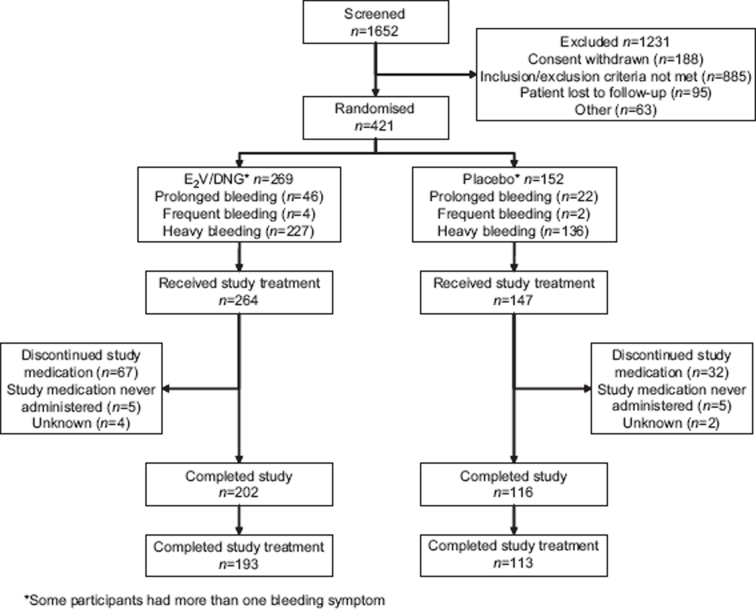
Pooled flow of participants through two studies, one conducted in North America and the other in Europe and Australia.

Across the two studies, 421 women were randomised to treatment and made up the ITT population (Figure 1). Of these, 411 women received at least one dose of study medication. The demographic and baseline characteristics of the ITT population are shown in Table 1. The majority of women enrolled in the two studies were Caucasian, although the proportion of Black women was higher in the North American study (52/190,27%) than in the European/Australian study (1/231, <1%).The most common symptom was heavy menstrual bleeding, followed by prolonged bleeding (Table 1). Frequent bleeding was rare across both studies.

**Table 1:** Demographic and baseline characteristics of women assigned to oestradiol valerate/dienogest (E_2_V/DNG) or placebo

	E_2_V/DNG(n = 269)	Placebo (n = 152)
Age, years	38.3±71	378±72
Ethnicity, *n* (%)		
Caucasian	215 (79.9)	126 (82.9)
Black	39 (14.5)	14 (9.2)
Hispanic	8 (3.0)	6 (3.9)
Asian	3(1.1)	3 (2.0)
Other	4(1.5)	3 (2.0)
Weight, kg	71.0±11.5	71.0±11.2
Body mass index, kg/m^2^	25.5±3.7	25.9±3.4
Bleeding symptoms		
Heavy bleeding	227 (84.4)	136 (89.5)
Prolonged bleeding	46 (171)	22 (14.5)
Frequent bleeding	4(1.5)	2 (1.3)

All data are presented as mean ± standard deviation unless otherwise stated.

### Changes in menstrual blood loss

**Figure 2:**
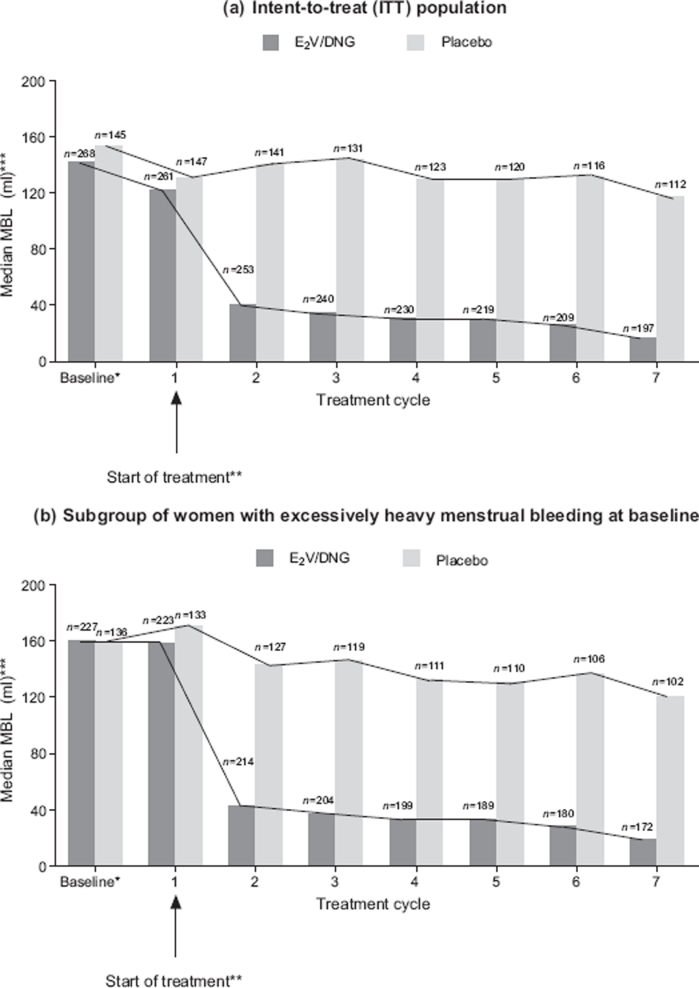
Median menstrual blood loss (MBL) by treatment cycle in patients treated with oestradiol valerate/dienogest (E_2_V/DNG) and placebo in (a) the intent-to-treat (ITT) population and (b) the subgroup of women with excessively heavy menstrual bleeding (defined as MBL greater than 80 ml at baseline; *n* = 227 for E_2_V/DNG and *n* = 136 for placebo). *For comparative purposes, baseline was calculated as (MBL volume during the 90-day run-in phase/90) × 28. **MBL observed during treatment cycle 1 represents the physiological menstrual bleeding (which triggers the start of treatment) plus any intermenstrual bleeding that may have occurred. ***Total blood loss during 28-day treatment cycles.

The changes in median MBL during the run-in phase and during active treatment are shown by treatment cycle in Figure 2 for both the ITT population and the subgroup of women with excessively heavy menstrual bleeding at baseline (MBL was determined from the collection of all sanitary items used over the course of each respective 28-day treatment cycle).

As study treatment was initiated on the first day of bleeding following randomisation, the bleeding observed at the start of the first treatment cycle essentially represents the natural bleeding that would otherwise have occurred without treatment; that is, patients would have been on treatment for only a few days at most. Since withdrawal bleeding usually spanned two treatment cycles (in approximately 60% of patients, the onset of withdrawal bleeding started from day 27 onwards [unpublished data]), MBL during any of the seven treatment cycles represents the latter part of ongoing withdrawal bleeding that started in a previous cycle, any additional intermenstrual bleeding episodes plus the start of the next withdrawal bleeding. Therefore, the MBL observed during treatment cycle 2, in large part, represents the first ‘on treatment’ withdrawal bleed.

In the ITT group, the reduction in MBL with E_2_V/DNG treatment was rapid, at the first withdrawal bleed after treatment initiation, and it was sustained over the duration of treatment; in fact, blood loss continued to decrease further over successive treatment cycles (Figure 2). After six months of treatment, i.e., by treatment cycle 7, the median MBL was reduced by 88%. In contrast, there was only a minor reduction in median MBL with placebo treatment: it had decreased by 24% by treatment cycle 7. For corresponding data in the subgroup of women with excessively heavy menstrual bleeding who received treatment with E_2_V/DNG, median MBL was also reduced by an overall 88% from the run-in phase to treatment cycle 7. The reduction in monthly MBL in women with excessively heavy menstrual bleeding treated with placebo was minimal: it had diminished by 23% by treatment cycle 7.

The mean absolute total reduction in 90-day MBL from baseline to the 90-day efficacy phase was 414 ± 373 ml with E_2_V/DNG and 109 ± 300 ml with placebo (*p* < 0.0001 adjusted mean difference E_2_V/DNG vs. placebo). Corresponding data in the subgroup of women with excessively heavy menstrual bleeding were 454 ± 375 ml with E_2_V/DNG and 118 ± 302 ml with placebo (*p* < 0.0001 adjusted mean difference E_2_V/DNG vs. placebo).

**Figure 3:**
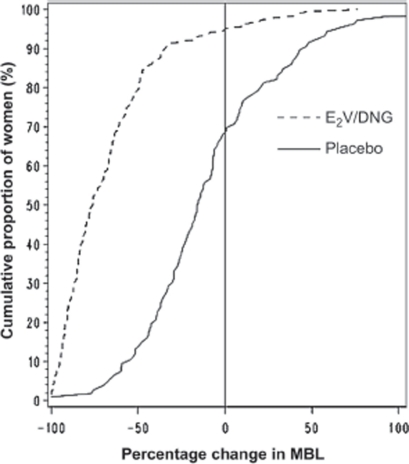
Percentage change in menstrual blood loss (MBL) volume from baseline (90-day run-in phase) to the efficacy phase (90-day efficacy phase) experienced by the proportion of women (intent-to-treat [ITT]).

Overall, a 20%, 50% and 80% reduction in MBL was achieved by approximately 92%, 80% and 46% of women in the E_2_V/DNG group, respectively; such reductions were only achieved by much smaller proportions of women in the placebo group (42%, 14% and 2%, respectively). An increase in total MBL volume during treatment (90-day efficacy phase) was observed in around 5% of E_2_V/DNG recipients and 30% of placebo recipients (Figure 3).

**Figure 4:**
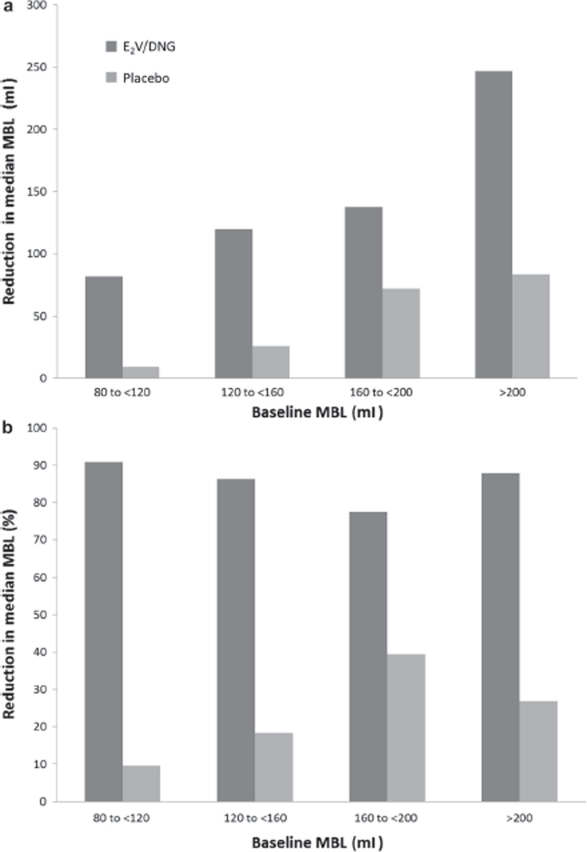
Absolute and relative reduction in menstrual blood loss (MBL) according to baseline MBL in the intent-to-treat (ITT) population. Data presented as (a) median reduction in MBL volume from baseline to treatment cycle 7 and (b) median percentage reduction from baseline to treatment cycle 7

Treatment with E_2_V/DNG was effective in reducing MBL irrespective of initial baseline MBL (Figure 4). Interestingly, although the percentage reduction in measured MBL appeared to augment with increasing baseline MBL in the group treated with placebo, this reduction remained relatively stable in the group treated with E_2_V/DNG (medians ranged from 77% to 91%).

### Number of sanitary protection items used

The observed reduction in MBL volume was associated with a decrease in the number of sanitary protection items used. The number of sanitary protection items used diminished from an average of 85.2 items during the run-in phase to 46.7 during the 90-day efficacy phase (mean reduction 40.6 ±35.0 items; median 40 items reduction from 79 to 38 items [range −215 to 58]) in women treated with E_2_V/DNG. In the placebo group, the corresponding decrease was from an average 88.6 items during the run-in phase to 66.7 during the 90-day efficacy phase (mean reduction 18.5 ± 37.3 items; median 12 items reduction from 81 to 62 items [range —256 to 31]). The mean between-treatment difference in sanitary protection items used was significantly in favour of E_2_V/DNG (−22.1 items [95% CI −30.7, −13.6; *p* < 0.0001]).

### Iron metabolism parameters

A minority of women used medications containing iron at some point during the study; overall, concomitant use of medications containing iron was reported in 45 of 269 women (16.7%) in the E_2_V/DNG group and in 39 of 152 women (25.7%) in the placebo group. Mean levels of haemoglobin, haematocrit and ferritin at baseline and on day 196 of treatment in women who received E_2_V/DNG or placebo are shown in Table 2. A marked significant improvement was observed in all measured iron metabolism parameters in women treated with E_2_V/DNG. In contrast, little or no improvement was observed in women who received placebo.

**Table 2:** Levels of haemoglobin, haematocrit and ferritin at baseline and day 196 of treatment in women who received oestradiol valerate/dienogest (E_2_V/DNG) or placebo

	E_2_V/DNG			Placebo		
		
	Baseline	Day 196	Change from baseline	Baseline	Day 196	Change from baseline
Haemoglobin, g/dl	*n* = 269	*n* = 245	*n* = 245	n=152	n=135	n=135
	12.2 ± 1.3	12.8 ± 1.1	+ 0.64±1.1***	12.0± 1.4	12.2 ± 1.3	+ 0.12 ± 1.0
Haematocrit, %	*n* = 269	*n* = 244	*n* = 244	*n* = 152	*n* = 135	*n* = 135
	38.7±3.8	40.1 ±3.7	+ 1.48±3.7**	38.5±4.3	38.7±4.0	+ 0.08±3.1
Ferritin, ng/ml	*n* = 269	*n* = 249	*n* = 249	*n* = 150	*n* = 137	*n* = 136
	179±25.9	25.5±24.4	+ 71 ±28.8*	172 ± 16.9	18.7±173	+1.2 ± 12.2

Data presented as mean ± standard deviation.

* *p*< 0.05 versus change from baseline with placebo

** *p* = 0.0002 versus change from baseline with placebo

*** *p*< 0.0001 versus change from baseline with placebo.

### Investigators' and patients' ratings of symptom improvement

The proportion of investigators who considered that their patients' bleeding symptoms at the end of cycle 7 had improved (including rating categories ‘much improved’ or ‘very much improved’) was significantly greater for patients treated with E_2_V/DNG (83.0%) than with placebo (40.6%; *p* < 0.0001). Similarly the proportion of patients who rated their bleeding symptoms as improved at the end of cycle 7 was also significantly greater in the E_2_V/DNG group (79.2%) than in the placebo group (42.4%;*p* < 0.0001).

### Safety

Safety outcomes were assessed in 264 women treated with E_2_V/DNG and 147 women treated with placebo. A total of 174 women (65.9%) treated with E_2_V/DNG and 85 women (57.8%) treated with placebo reported at least one adverse event. Adverse events reported in at least 2% of women are shown in Table 3. The majority of adverse events that occurred in both treatment groups were mild or moderate in intensity. Overall, 26 women (9.8%) treated with E_2_V/DNG and 9 women (6.1%) treated with placebo prematurely discontinued treatment because of adverse events.

**Table 3:** Adverse events that occurred in at least 2% of women who received oestradiol valerate/dienogest (E_2_V/DNG) or placebo (shown in alphabetical order)

Adverse events, n (%)	E_2_V/DNG (n = 264)	Placebo (n= 147)
Acne	10 (3.8)	3 (2.0)
Anaemia	4(1.5)	5 (3.4)
Anxiety	1 (0.4)	3 (2.0)
Arthralgia	3 (1.1)	4 (2.7)
Back pain	6 (2.3)	7 (4.8)
Breast pain	13 (4.9)	0 (0.0)
Breast tenderness	10 (3.8)	4 (2.7)
Bronchitis	6 (2.3)	2 (1.4)
Diarrhoea	5(1.9)	3 (2.0)
Dysmenorrhoea	7 (2.7)	4 (2.7)
Fatigue	8 (3.0)	4 (2.7)
Headache	26 (9.8)	20 (13.6)
Hypertension	5(1.9)	3 (2.0)
Influenza	6 (2.3)	1 (0.7)
Menorrhagia	3 (1.1)	4 (2.7)
Metrorrhagia	8 (3.0)	1 (0.7)
Migraine	6 (2.3)	2 (1.4)
Nasopharyngitis	21 (8.0)	10 (6.8)
Nausea	13 (4.9)	6 (4.1)
Pharyngitis	1 (0.4)	4 (2.7)
Serum ferritin decreased	3 (1.1)	6(4.1)
Sinusitis	5(1.9)	3 (2.0)
Vaginosis bacterial	7 (2.7)	4 (2.7)
Vertigo	0 (0.0)	3 (2.0)
Viral infection	6 (2.3)	1 (0.7)
Vomiting	5(1.9)	6 (4.1)
Vulvovaginal mycotic infection	5(1.9)	3 (2.0)
Weight gain	9 (3.4)	1 (0.7)
γ-glutamyltransferase increased	3 (1.1)	3 (2.0)

Treatment-related adverse events (as determined by the treating physician) were reported more often in women treated with E_2_V/DNG than placebo (*n* = 98 [37.1%] vs. *n* = 25 [17.0%]).Treatment-related adverse events occurring in at least 2% of women in either treatment group included headache (E_2_V/DNG, 6.4% vs. placebo, 6.1%), breast pam (4.5% vs. 0.0%), acne (3.4% vs. 2.0%),breast tenderness (3.0% vs. 1.4%), metrorrhagia (irregular menstrual bleeding: 3.0% vs. 0.7%), nausea (3.0% vs. 1.4%), dysmenorrhoea (overall incidence: 2.7% vs. 0.0%), and weight gain (2.3% vs. 0.0%).

Serious adverse events were rare; they were reported by three women (1.1%) treated with E_2_V/ DNG (myocardial infarction, *n* = 1; chronic cholecystitis, *n* = 1; breast cancer *in situ,* *n* = 1) and four women (2.7%) treated with placebo (vertigo, *n* = 1; chest pain, *n* = 1; spontaneous abortion, *n* = 1; suicide attempt, *n* = 1). The myocardial infarction supervened in a 46-year-old woman who had a history of hyperlipidaemia and a family history of cardiovascular disease, two days after she had taken the last dose of study medication. This event was considered to be possibly related to treatment. The case of breast cancer *in situ,* a 4 cm lesion, was diagnosed five months after initiating treatment in a women aged 45 years. This event was also considered to be possibly related to treatment. The case of chronic cholecystitis occurred after 22 days of study treatment in a 37-year-old woman. All three of the patients who suffered the serious adverse events reported (i.e., myocardial infarction, breast cancer and chronic cholecystitis) recovered. No deaths were reported in these studies.

## DISCUSSION

This pooled analysis shows that E_2_V/DNG rapidly reduces MBL in women with heavy and/or prolonged menstrual bleeding. This effect is achieved at the first withdrawal bleed after treatment initiation and is sustained over the duration of treatment. By pooling data from the two Phase III trials conducted in Europe and Australia and North America, the decrease in MBL achieved with E_2_V/DNG was shown to be consistent across a larger and more diverse population of women. Moreover, the reduction in MBL was associated with a decrease in the number of sanitary protection items used and an improvement in iron metabolism parameters.

In the present study, the percentage reduction in MBL with E_2_V/DNG appears to be independent of baseline MBL. Interestingly, small reductions in MBL were also observed in the placebo group, with higher percentage reductions observed among those with highest baseline MBL. It may be postulated that the willingness to continue to adhere to study procedures (such as the highly demanding process of collecting used sanitary items) may have been compromised if those receiving treatment perceived a lack of clinical efficacy and, therefore, became less motivated to participate fully. This lack of motivation to fully participate would likely disproportionately affect the placebo group, especially those with highest baseline MBL, and, as such, would have affected the total count of sanitary items used, leading to an underestimation of blood loss determination by using the alkaline haematin method.

Although not directly comparable, the median decrease in MBL achieved by treatment cycle 7 with E_2_V/DNG treatment (88%) appears to approach that achieved with the LNG-IUS (median 95% and 96% reduction) over six cycles in two studies that also used the alkaline haematin method to objectively assess blood loss in women with heavy menstrual bleeding^15^,^16^. The LNG-IUS (*n* = 82 and *n* = 25, respectively) produced significantly greater reductions in median monthly MBL than the active comparators used: short-course oral medroxyprogesterone (*n* = 83) 10 mg daily for ten days starting on the 16th day of the menstrual cycle (21%)^15^ or mefenamic acid (*n* = 26) 500 mg three times daily for the first four days of the menstrual cycle (17%)^16^. By comparison, a median 24% reduction in MBL by treatment cycle 7 occurred in the placebo group in our study. Placebo-related reductions in measured blood loss have been recorded in heavy menstrual bleeding in a previous study over six menstrual cycles^17^.

Oral tranexamic acid (1.3 g three times daily for five days from the start of menstruation) was shown to reduce mean MBL by 40.4% (vs. 8.2% reduction with placebo) in a recent study that also used the alkaline haematin method to objectively assess blood loss^17^.These results with oral tranexamic acid appear rather modest in comparison with the reductions in blood loss achieved with E_2_V/DNG in our pooled analysis. Additionally, the added advantage of using E_2_V/DNG is that it provides effective contraceptive protection and use of back-up contraception is not required. Agents such as tranexamic acid do not offer contraceptive protection and should not be used in women using COCs^18^. Similarly, cyclical progestogens do not provide adequate contraceptive protection and additional non-hormonal backup contraception would be also required.

E_2_V/DNG represents the first COC approved for the treatment of heavy and/or prolonged menstrual bleeding in many countries worldwide. Other oral contraceptives have not been formally approved for the treatment of heavy menstrual bleeding but, have nonetheless, been frequently used off-label with some success.The available studies assessing the effectiveness of other COCs in reducing MBL in women with heavy menstrual bleeding are limited to three randomised comparative studies with the LNG-IUS^19^–^21^, one of which was in women with fibroid-related heavy menstrual bleeding^20^, and a randomised crossover study that included mefenamic acid, naproxen and danazol^7^.

In the first comparative study^21^, which used the semi-quantitative pictorial blood loss assessment chart (PBAC) to assess MBL, treatment with a COC containing norethindrone acetate (NETA) 1 mg and ethmylestradiol (EE) 20 µg (21/7 regimen; *n* = 19) resulted in a 68% reduction in median MBL scores at 12 months (vs. 83% with the LNG-IUS; between-group differences *p* = 0.002). In the second comparative study^19^, treatment with a COC containing LNG 150 µg/EE 30 µg (21/7 regimen; *n* = 56) resulted in a mean reduction in MBL at 12 months of 35%, as assessed using the alkaline haematin method (vs. 87% with the LNG-IUS [*n* = 56]; *p* = 0.013).PBAC scores were also used in this trial and suggested a 2.5% reduction in scores with the COC (vs. 87% with the LNG-IUS; *p* < 0.001). The discrepancy between MBL measured by the alkaline haematin method and the PBAC score may be due, in part, to the inherent wider variability between PBAC scores and measured MBL with increased volume of blood loss. The study by Higham *et al.* also shows the limitations of the PBAC method for the determination of MBL and supports the validity of the methodology used in the present study^22^. In the study of women with fibroid-related heavy menstrual bleeding^20^, the mean reduction at 12 months in MBL with the COC (*n* = 29), also assessed using the alkaline haematin method, was 13% (vs. 91% with the LNG-IUS; *p* < 0.001). Pictorial blood loss assessment chart scores corresponded to a 54% reduction in scores (vs. 88% with the LNG-IUS; *p* = 0.02).

In the randomised cross-over study that included mefenamic acid, naproxen and danazol^7^, treatment with a COC containing LNG 150 Hg/EE 30 µg (21/7 regimen; *n* = 12) for two cycles resulted in a 43% decrease in mean MBL compared with control cycles (assessed using the alkaline haematin method). In comparison, mefenamic acid, naproxen and danazol reduced mean MBL by 20–39%, 12% and 49%, respectively. There was no significant difference between the COC, mefenamic acid, low-dose danazol or naproxen treated groups.

The reduction in MBL achieved with E_2_V/DNG in the present study was accompanied by significant rises in haemoglobin, haematocrit and ferritin levels from baseline, which were significantly greater than with placebo. Although some improvements were observed in the placebo group, these were not significant and may, in part, have been due to the use of iron supplements in a minority of patients during the study. Similarly, treatment with the COC containing NETA 1 mg/EE 20 µg (21/7 regimen) or the LNG-IUS increased mean haemoglobin levels significantly from baseline to 12 months (*p*<0.001) in women with idiopathic heavy bleeding^21^. Interestingly, the raised haemoglobin levels plateaued after six months of treatment with the LNG-IUS, while the increase observed with the COC occurred at a much slower rate, progressing linearly with time over the 12 months. In contrast, in the two studies comparing the LNG-IUS with a COC containing LNG 150 µg/EE 30 µg (21/7 regimen) over 12 months, haemoglobin and ferritin levels significantly increased with the LNG-IUS, but not with the COC^19^,^20^. No improvements in haemoglobin and ferritin levels were reported in a placebo-controlled study assessing tranexamic acid (*n* = 123) over six cycles of treatment despite significant reductions in MBL^17^.

E_2_V/DNG represents a valid alternative to current treatments for heavy menstrual bleeding. The consideration of contraindications and precautions for use of any individual treatment option, including E_2_V/DNG, is warranted to ensure a positive benefit-risk balance. The side effect profile of COCs is well established and includes an increased risk for thrombosis which must be carefully balanced against the benefits achieved. Hysterectomy and endometrial ablation are effective treatments, but are associated with surgical risks and are not suitable for women who wish to retain their fertility. Other approved medical therapies, apart from the LNG-IUS, do not provide effective contraceptive protection and use of back-up contraception would be required. However, active comparator studies with E_2_V/DNG in the treatment of heavy and/or prolonged menstrual bleeding are currently lacking.

No specific safety concerns were noted with E_2_V/DNG treatment across the two studies pooled. The type and frequency of adverse events reported with E_2_V/DNG were typical of hormonal contraceptive use.

In conclusion, E_2_V/DNG rapidly reduces MBL in women with heavy and/or prolonged menstrual bleeding. This effect is achieved at the first withdrawal bleed after treatment initiation and is sustained for the duration of treatment, irrespective of initial MBL.
